# T‐cell activation and immune memory enhancement induced by irreversible electroporation in pancreatic cancer

**DOI:** 10.1002/ctm2.39

**Published:** 2020-06-04

**Authors:** Chaobin He, Xin Huang, Yu Zhang, Xiaojun Lin, Shengping Li

**Affiliations:** ^1^ Department of Pancreatobiliary Surgery State Key Laboratory of Oncology in South China Collaborative Innovation Center for Cancer Medicine Sun Yat‐sen University Cancer Center Guangzhou P. R. China; ^2^ State Key Laboratory of Ophthalmology Zhongshan Ophthalmic Center Sun Yat‐sen University, Guangzhou Guangdong P. R. China

**Keywords:** immune memory, irreversible electroporation, pancreatic ductal adenocarcinoma cancer, T cell

## Abstract

**Background:**

Irreversible electroporation is shown to induce immune changes in pancreatic cancer while the histology evidences are still lacking. The aim of this study is to show the immune changes in histology and explore whether irreversible electroporation (IRE) can induce immunogenic cell death (ICD) of tumor cells and activate specific immune responses.

**Methods:**

Subcutaneous and orthotopic pancreatic cancer models were established and used to evaluate the effect of immune modulation of IRE. The infiltration of T cells was assessed in several tissue samples before and after IRE. Abscopal effect was then assessed by comparing the tumor growth of subcutaneous tumors after in situ ablation with IRE or exposure to tumor culture supernatant (TSN) of IRE‐treated Pan02. The expression of damage‐associated molecular patterns (DAMPs) of tumor cells after IRE was detected in vitro.

**Results:**

IRE could significantly suppress the tumor growth and increase the infiltration of CD8^+^ T cells. After ablation with IRE or stimulation with TSN of Pan02 treated by IRE, the growth of untreated tumor was suppressed and the effector CD8^+^ T cells and memory T cells increased significantly in mice. Additionally, the inhibition effect of tumor growth increased along with the increasing strength levels of electroporation. IRE induced ICD of tumor cells by increasing the synthesis and secretion of DAMPs.

**Conclusions:**

IRE induced local immunomodulation by increasing specific T cells infiltration. Through enhancing specific immune memory, IRE not only led a complete tumor regression in suit, but also induced abscopal effect, suppressing the growth of the latent lesions.

## Graphical abstract

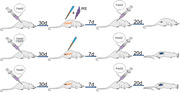

**Abbreviations**
PDAC: pancreatic ductal adenocarcinoma cancer
Treg: regulatory T cell
MDSC: myeloid derived suppressor cell
TME: tumor microenvironment
CAF: cancer‐associated fibroblast
IRE: irreversible electroporation
DAMP: damage‐associated molecular pattern
HMGB1: high‐mobility group B1
HSP: heat shock protein
ICD: immunogenic cell death
FBS: fetal bovine serum
PBS: phosphate‐buffered saline
IHC: immunohistochemistry
IHF: immunohistofluorescence
LN: Iymph node
MVD: microvessel density
APC: antigen‐presenting cell

## 1 BACKGROUND

Pancreatic ductal adenocarcinoma cancer (PDAC) is a highly lethal and refractory malignancy with extremely poor survival.^1^ Difficulty in early diagnosis and early tumor metastasis has led to the low resection rates^2^ and poor survival.^3^ Palliative chemotherapy is the first choice for most PDAC. However, the response rates for chemotherapy are low.^4^ Immune checkpoint inhibitors, which have achieved many successes in other kinds of tumors, also demonstrated disappointing results in PDAC.^5^, ^6^ PDAC has an immunosuppressive nature, which is characterized by the enrichment of fibrotic stroma and infiltration of suppressive immune cells, including tumor‐associated macrophages, regulatory T cells (Tregs), and myeloid‐derived suppressor cells (MDSCs), and further limits the efficacy of chemotherapy and immune checkpoint inhibitors.^7^

More than closed relationship between PDAC stroma and immune cells, cross‐talk is observed among immune cells in the tumor microenvironment (TME) of PDAC. MDSCs are able to attract Treg cells in a cell‐cell dependent manner while, Treg cells affect the survival and the proliferation of MDSCs.^8^ Moreover, the interactions between MDSCs and Treg cells contribute to the formation of fibrotic stroma, which can physically exclude cytotoxic T cells from the vicinity of tumor cells.^8^, ^9^ The interactions between stroma and immune‐suppresive cells contribute to the immune‐suppressive TME of PDAC. Some studies that focused on the modulation of PDAC stroma have resulted in different results. The depletion of fibroblast activation protein alpha‐positive cancer‐associated fibroblasts (CAFs) improved the efficacy of anti‐PDL1 blockade.^10^ Oppositely, depletion of alpha smooth muscle actin‐positive CAFs promoted the infiltration of Tregs and produced an alarmingly aggressive phenotype of PDAC.^11^ Additionally, stroma was shown to able restrain PDAC from an unchecked growth.^12^ Considering these results, it is indicated that maybe modulation of stroma via a local approach while preserving the tumor restraining matrix may provide new insights to the treatment of PDAC.

Irreversible electroporation (IRE) is a newly developed nonthermal ablative technology that produces an extremely high electric field across cells, leading to cell membrane disruption and cell apoptosis.^13^ As an effective method, IRE provides patients with locally advanced pancreatic cancer (LAPC) with encouraging survival results.^14^, ^15^, ^16^ Compared to other thermal ablative methods, IRE owns the nonthermal feature, which ensures the clinical effect is free of heat sink effect and leaves the supporting tissue largely unaffected.^17^, ^18^ Previous studies had shown that thermal ablation induces systemic anti‐tumor immune.^19^, ^20^ Considering the nature of preservation of vessels, which is helpful for the transmission of immune molecules or cells, IRE may be more immunological sensitive than thermal ablations. Robert et al had shown that IRE therapy had greater therapeutic efficacy in immunocompetent mice than what had been suggested by immunodeficient models, indicating that IRE could invoke a systemic response beyond the targeted ablation region.^21^ A few studies have evaluated the immune responses of IRE while most of these studies were based on the changes of immune cells in peripheral blood of PDAC patients after IRE.^22‐24^ Other in vivo studies illustrating the immune changes following IRE were mostly based on subcutaneous models,^25^, ^‐^, ^27^ which might lead to a lack of the simulation of the main characteristics of stroma in PDAC. Although IRE is shown to indicate a possible role in the host immune system, there are no evidences of stroma modulation and immune cells changes in the poorly immunological orthotopic PDAC.

**HIGHLIGHTS**

1. Abscopal effect could be induced by IRE in the treatment of pancreatic cancer.
2. IRE increased specific T‐cells infiltration and immune response.
3. IRE suppressed the growth of the latent lesions through enhancing specific immune memory.

Nowadays, it has become clear that antitumor therapy acts more successfully if it can kill tumor cell directly, along with inducing an immunogenic form of cell death.^28^ Through regulating emission of damage‐associated molecular patterns (DAMPs), including high‐mobility group B1 (HMGB1), calreticulin and heat shock protein (HSP), and immunogenic cell death (ICD) contributes to the activation of an immune response specific for cancer cells.^29^, ^30^ Due to the characteristics of inducing cell apoptosis, some molecule patterns are released from the dying or dead cells following IRE, which may act as specific antigens, contribute to the activation of T cells, and induce specific immune responses.

Based on these analyses, we speculate that apart from local tumor ablation, IRE can lead to both local and systemic control of disease through the priming or boosting immune response specific for against tumor. In the present study, we aim to evidence the immune changes following IRE and evaluate the immune effect as a tumor‐specific vaccination based on the orthotopic and subcutaneous models of PDAC.

## 2 METHODS

### 2.1 Cell lines and animal models

Human and murine pancreatic adenocarcinoma cell lines Panc‐1 andPan02 were purchased from Cell Bank of the Chinese Academy of Sciences (Shanghai, China). Cells were maintained at 37°C in a humidified incubator and 5% CO_2_ atmosphere in DMEM or RPMI 1640 medium supplemented with 10% heat‐inactivated fetal bovine serum (FBS) (Gibco, Los Angeles, California, 90001, USA) and 1% of penicillin‐streptomycin (10 000 U/mL; Life Technology, Los Angeles, California, 90001, USA). The cells were tested every 2 months for mycoplasma contamination.

All animal studies complied with relevant ethical regulations for animal testing and research, and were approved by the Institutional Animal Care and Use Committee of Sun Yat‐sen University Cancer Center. Animals were maintained and studies were carried out in accordance with institutional guidelines. Subcutaneous pancreatic cancer model was established by subcutaneous injection of 6 × 10^6^ Pan02 cells into the left back of 6‐week‐old C57BL/6 mice. Following the instruction of previous study,^31^ the orthotopic pancreatic cancer model was established by injecting 6 × 10^6^ Pan02 cells into the parenchyma of the pancreas through a small abdominal incision. The needle was removed 10 seconds after completion of the injection, and the incision was closed with absorbable sutures.

### 2.2 Electroporation

Electroporation was performed when tumor reached 7‐8 mm diameter (8‐10 days after implantation) using an ECM 830 square wave pulse electroporator (BTX Harvard Apparatus, Holliston, Massachusetts, 01746, USA). Mouse were anesthetized with 2% isoflurane in 3 L/min oxygen flow and injected percutaneously with buprenorphine analgesic (0.1 mg/kg). Through puncturing the skin above the tumor or a small abdominal incision, the subcutaneous or orthotopic tumor was bracketed along the long axis using the two‐needle probe with a 5 mm gap followed by the delivery of electric pulses. The electric array fully penetrated the whole tumor to maximize the effect of electroporation, with the following parameters: voltage: 1000 V; pulse duration: 100 ms; pulse frequency: 1 Hz; pulse number: 80. For control, surgical procedures and needles placement were performed to tumors without electric pulses using the same anesthetic conditions.

For electroporation experiments of cells in vitro, tumor cells were trypsinized, resuspended in phosphate‐buffered saline (PBS) at 2 × 10^6^ cells mL^−1^, and added to an electroporation cuvette (1652088; BTX, Holliston, Massachusetts, 01746, USA) embedded with two aluminum plate electrodes 4 mm apart. The cell suspension was in direct contact with the plate electrodes, and subjected to electroporation at room temperature with the following parameters: voltage: 200–600 V; pulse duration: 100 µs; pulse repetition frequency: 1 Hz; number of repetition pulses: 20. The parameters were in consistent with clinically used values.^32^, ^33^ After electroporation, the cell suspension was kept on ice and analyzed or used within 30 minutes. Similar with other study,^34^ tumor culture supernatant (TSN) was collected after specific cells were cultured for 3 days after electroporation with different strength levels and the cell debris was filtered. Three independent repetitions were performed for each in vitro experiment.

### 2.3 Cell viability assay

Tumor cell viability after IRE treatment was detected as previously described using Cell Counting Kit 8 (CCK‐8; CK04; Dojindo Laboratories, Kumamoto, Japan).^35^

### 2.4 Western blot analysis

Western blot analysis was performed as previously described.^35^ The following antibodies were used: anti‐HMGB1 (1:1000, ab79823; Abcam, Cambridge, UK), anti‐HSP70 (1:1000, ab181606; Abcam), anti‐calreticulin (1:1000, ab92516; Abcam), and anti‐GAPDH (1:1000, 60004‐1‐Ig; Proteintech，Chicago, Illinois, 60601, USA). Proteins were visualized by using an ECL kit (4AW011; purchased from 4A Biotech Co., Ltd, Beijing, China). The integrated optical density of the protein bands was determined using Image Lab 4.0 image acquisition and analysis system software. Simultaneous detection of GAPDH protein bands was used as the internal reference.

### 2.5 Enzyme‐linked immunosorbent assay

The supernatant of treated cells was collected and centrifuged at 12 000 × *g* for 1 minute. HMGB1 levels in the media were analyzed using enzyme‐linked immunosorbent assay (ELISA) (JYM0485Mo and JYM0485Hu; Jiyinmei, Wuhan, China) as described by the manufacturer.

### 2.6 Immunohistochemistry and immunohistofluorescence

Mouse tumors and relative organs were harvested, fixed in formalin, and embedded in paraffin before being cut into 4 µm sections. Paraffin‐embedded tissue sections were deparaffinized by xylene and rehydrated by graded ethanol dilutions. For antigen retrieval, tissue sections were pressure cooker for 3 minutes in EDTA (pH 8.0) and were blocked in 3% BSA‐containing PBS for 30 minutes at room temperature. For tissue staining, the tissue sections were incubated with primary antibodies overnight at 4°C. The primary antibodies include anti‐mouse CD3 (ab231830; Abcam), anti‐mouse CD8 (ab209775; Abcam), anti‐mouse CD4 (ab183685; Abcam), anti‐mouse CD31 (ab1829181; Abcam), anti‐mouse lysyloxidase (LOX, ab221936; Abcam).

For immunohistochemistry (IHC), the 3,3′‐diaminobenzidine (DAB) system was used to visualize staining. Tissue sections were washed with PBS plus 0.1% Tween‐20, and then incubated with biotinylated secondary antibody and streptavidin‐conjugated horseradish peroxidase (DAKO; Carpinteria, California, 93013, USA) for 30 minutes each. A positive reaction was detected by exposure to DAB system. Slides were counterstained with hematoxylin and visualized under a bright‐field microscope at 40× and 400× magnification.

For immunohistofluorescence (IHF), tissue sections were incubated with Alexa Fluor 488‐conjugated anti‐goat IgG (A11008; Invitrogen, Carlsbad, California, 92101, USA) or 594‐conjugated anti‐goat IgG (A11005; Invitrogen) at 37°C for 1 hour. Nuclei were counterstained with DAPI. Immunofluorescence staining images were taken by ZEISS microscope (LSM880; Jena, Germany). Positive cells were quantified using ImagePro Plus software (Media Cybernetics, Annapolis, Maryland, 21401, USA) and expressed as mean ± SEM in high‐powered fields detected by confocal microscopy.

### 2.7 Analysis of tumor‐infiltrated immune cells

Mice bearing subcutaneous and orthotopic tumors were euthanized 7 days after IRE, and tumors were harvested and dissociated using a mouse tumor dissociation kit according to manufacturer's recommendations (Miltenyi Biotec,Kreis, Germany). Single cell suspensions were available after tumor cells were passed through a 70 µm strainer and then stained with antigen‐presenting cell (APC)‐conjugated anti‐mouse CD8 (100712; Biolegend, San Diego, California, 92101, USA), FITC‐conjugated anti‐mouse CD4 (100406; Biolegend), PE‐conjugated anti‐mouse CD3 (100206; Biolegend), APC/Cyanine7‐conjugated anti‐mouse CD8 (100714; Biolegend), FITC‐conjugated anti‐mouse/human CD44 (103006; Biolegend), and PE/Cy7‐conjugated anti‐mouse CD62L (104417, Biolegend), respectively, on ice for 15 minutes (3 × 10^6^ cells/sample). The samples were washed for three times and resuspended in 200 µL of cold PBS containing 2% FBS and 1 mM EDTA for analysis using flow cytometry (FC; CytoFLEX, Beckman Coulter, Brea, California, 92821, USA). The gating strategy is shown in Figure S1.

### 2.8 Statistical analysis

Statistical analysis was carried out using GraphPad Prism 8.0 software (GraphPad Software Inc., San Diego, , California, 92101, USA). Values are mean ± standard error of the mean (SEM). Statistical differences between groups were calculated either using the Student's *t*‐test or analysis of variance (ANOVA) with post hoc multiple comparisons. The log‐rank test was used in Kaplan‐Meier survival analysis. A *P* value of <.05 was considered statistically significant.

## 3 RESULTS

### 3.1 The killing effect of IRE on pancreatic cancer cells

**FIGURE 1:**
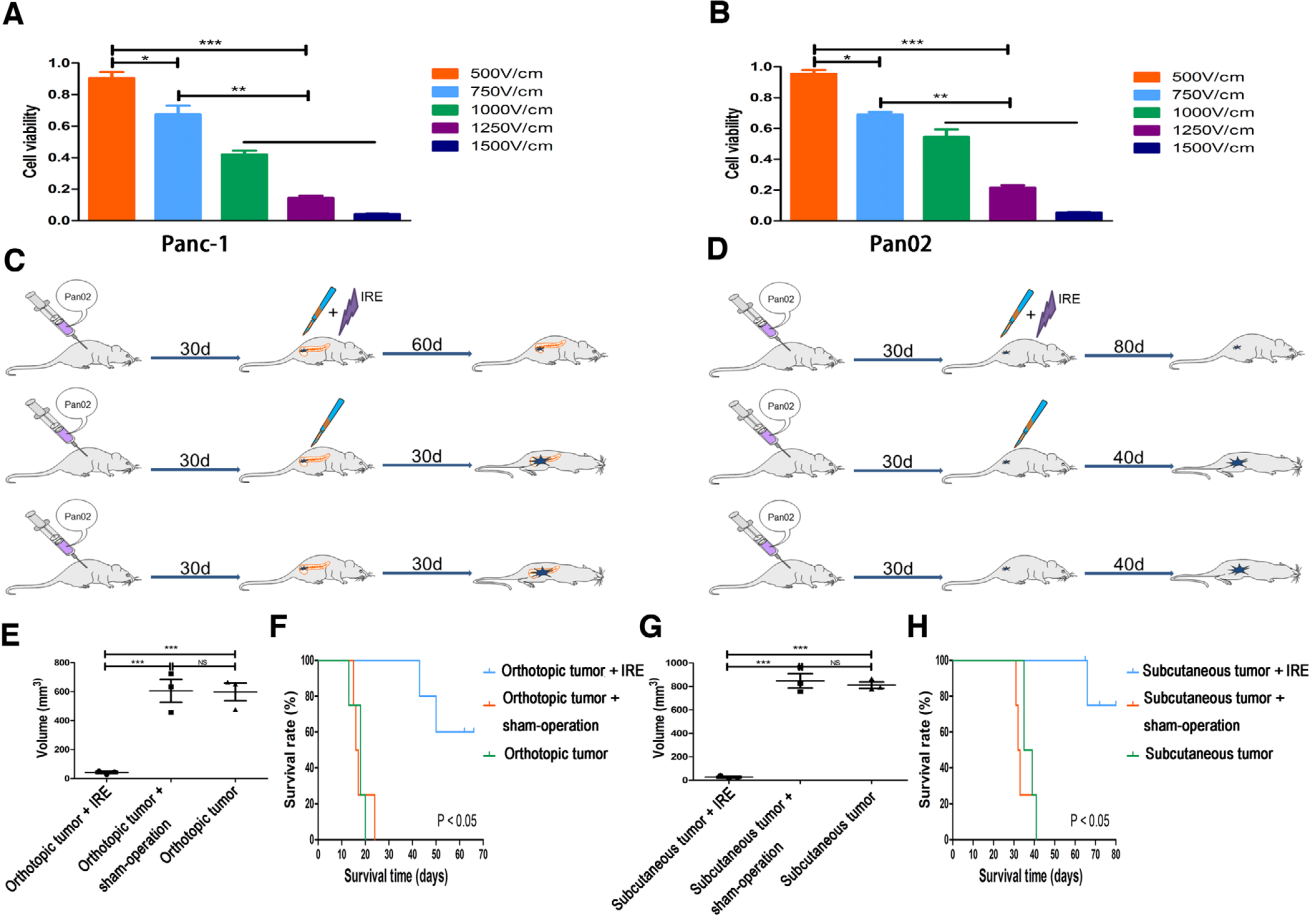
The effects of electric fields on viability of tumor cells. A, The effect of electric fields with different field strengths on viability of different kinds of Panc‐1 cells. The cell viability decreased gradually alone with the increasing electric field strengths. B, The effect of electric fields with different field strengths on viability of different kinds of Bxpc‐3 cells. The cell viability decreased gradually alone with the increasing electric field strengths. C, Experimental protocol for the evaluation of the effect of IRE in orthotopic pancreatic cancer model. D, Experimental protocol for the evaluation of the effect of IRE in subcutaneous pancreatic cancer model. E, The comparisons of tumor growth curves of orthotopic pancreatic tumor with or without IRE treatment. F, The comparisons of survival curves of orthotopic pancreatic tumor with or without IRE treatment. G, The comparisons of tumor growth curves of subcutaneous pancreatic tumor with or without IRE treatment. H, The comparisons of survival curves of subcutaneous pancreatic tumor with or without IRE treatment. One‐way analysis of variance (ANOVA) with Bonferroni comparison test was performed. **P < .01, ***P < .001, NS, not significant

Based on the electric field applied to tumor cells, IRE can induce cells apoptosis. To detect the killing effect of IRE on tumor cells, a CCK8 analysis was applied immediately after tumor cells were exposed to electroporation at different field strength levels. It was shown that cell viability decreased gradually alone with the increasing electric field strengths. At the electric field level of 1500 V/cm of electroporation that was regarded as IRE, cell viability decreased by more than 98% compared with the control group (Figure 1A,B). In addition, the antitumor efficacy of IRE was evaluated in both orthotopic and subcutaneous pancreatic cancer models. IRE, sham‐operation, and no treatment were performed in mice when tumors reached diameter of 7–8 mm (Figure 1C,D). Compared with mice in the no‐treatment and sham‐operation groups, mice in the IRE group had significantly smaller tumors in orthotopic pancreatic tumor models (Figure 1E). Furthermore, significantly longer survival time was also observed in mice in IRE group among all groups (Figure 1F). Similar results were obtained in subcutaneous tumor models (Figure 1G,H). IRE can significantly inhibit tumor growth in vitro and in vivo.

### 3.2 The increasing infiltrated CD8^+^ T cell contributed to the inhibition of tumor growth

**FIGURE 2:**
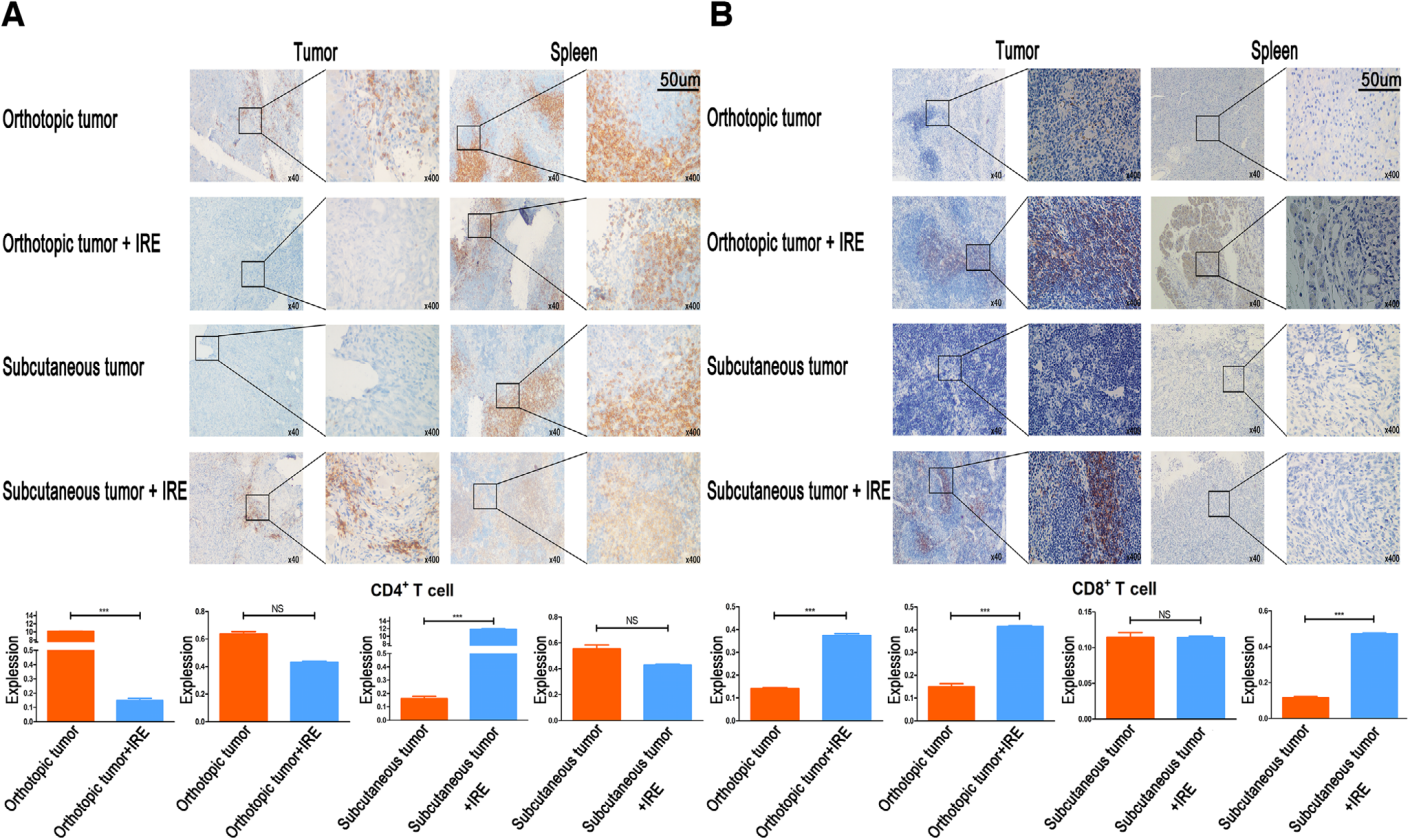
IRE could increase infiltration of CD8^+^ T cells in pancreatic tumor tissue. A, Representative image of independent tissue staining for IHC for CD4 (blue, nucleus; brown, antigen) and quantification of lymphocytes density of untreated control and IRE‐treated tumor or spleen in orthotopic and subcutaneous tumor models. Significant decrease and increase of CD4 in tumor of the orthotopic and subcutaneous tumor models, respectively, were observed after IRE. All images taken at 40× and 400× magnification; scale bar 50 µm. B, Representative image of independent tissue staining for IHC for CD8 (blue, nucleus; brown, antigen) and quantification of lymphocytes density of untreated control and IRE‐treated tumor or spleen in orthotopic and subcutaneous tumor models. Enhanced immune infiltration and a proinflammatory microenvironment following IRE are evident from increased CD8^+^ T cell in tumor and spleen of the orthotopic tumor model, and spleen of the subcutaneous tumor model. All images taken at 40× and 400× magnification; scale bar 50 µm. Two‐tailed Student's *t* test was performed. ^***^ *P* < .001, NS, not significant

**FIGURE 3:**
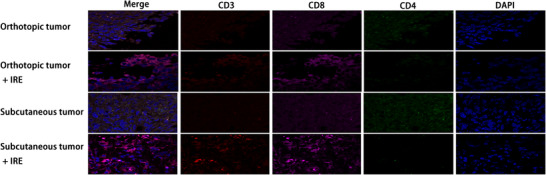
Representative image of independent tissue staining for IHF for CD4 and CD8 of untreated control and IRE‐treated tumors in orthotopic and subcutaneous tumor models. Increased CD8^+^ T cell in tumor following IRE in orthotopic and subcutaneous tumor models were observed while the changes in numbers of CD4^+^ T cell were not obvious

As IRE can generate durable antitumor responses in orthotopic and subcutaneous models, we then profile immune T cells in pancreatic tumor 7 days after IRE treatment. As detected by IHC (Figure 2) and IHF (Figure 3), significantly more proliferating CD8^+^ T cells infiltrated the tumor and spleen following IRE compared with control group. There were no significant differences in frequencies of CD4^+^ T cells between the IRE and control groups.

**FIGURE 4:**
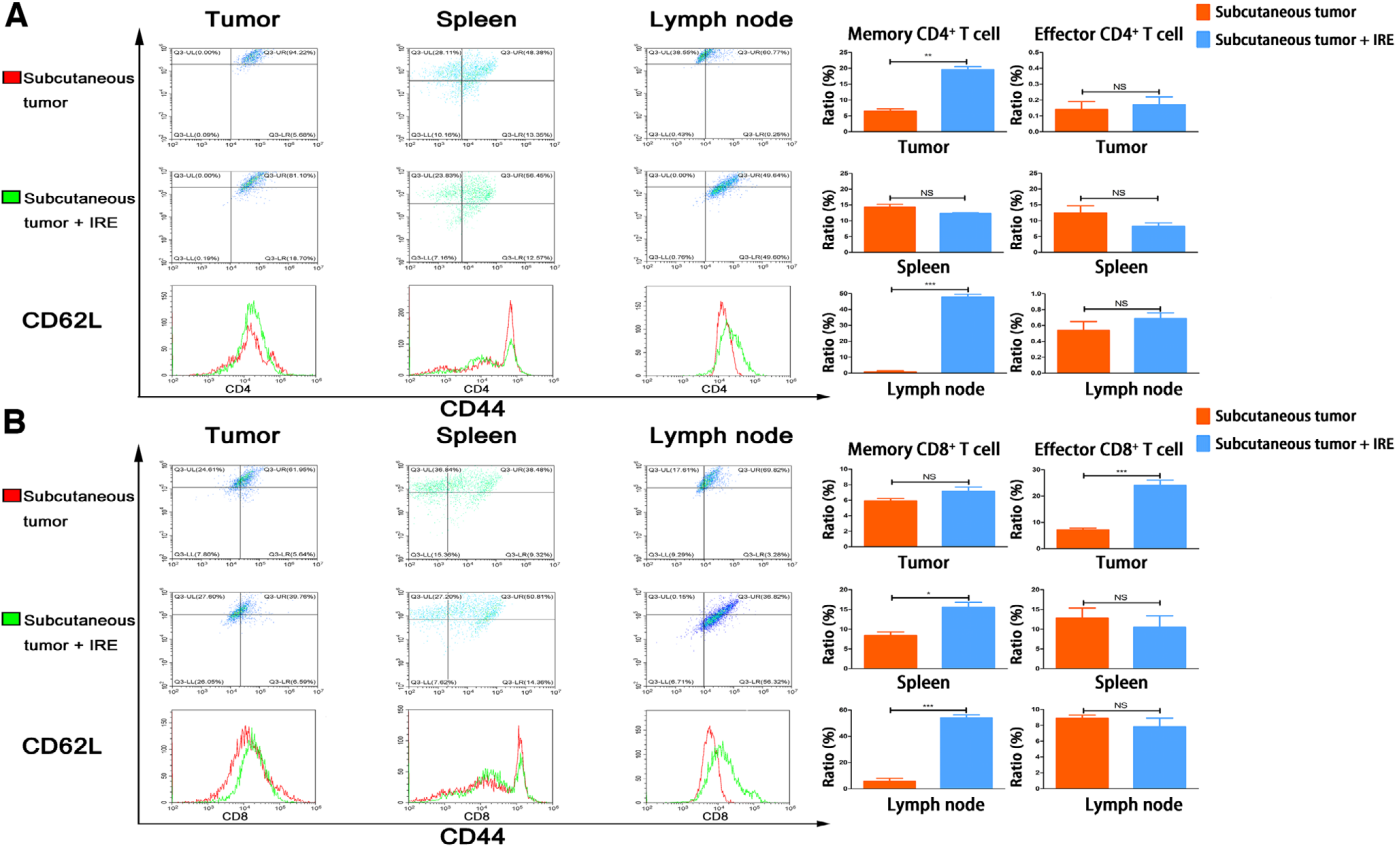
IRE increased the infiltration of memory CD4^+^ and effector CD8^+^ T cells in pancreatic tumor tissue. A, Analysis of T cells isolated from tumor, spleen, and lymph node and the comparisons of associated ratios of memory and effector CD4^+^ T cells in subcutaneous tumor models before and after IRE treatment. Memory CD4^+^ T cells increased significantly after IRE in tumor and lymph node, while no significant differences were observed in other groups. B, Analysis of T cells isolated from tumor, spleen, and lymph node, and the comparisons of associated ratios of memory and effector CD8^+^ T cells in subcutaneous tumor models before and after IRE treatment. Memory CD8^+^ T cells in spleen, lymph node, and effector CD8^+^ T cells in tumor increased significantly after IRE. Two‐tailed Student's *t*‐test was performed. *P < .05, **P < .01, ***P < .001, NS, not significant

**FIGURE 5:**
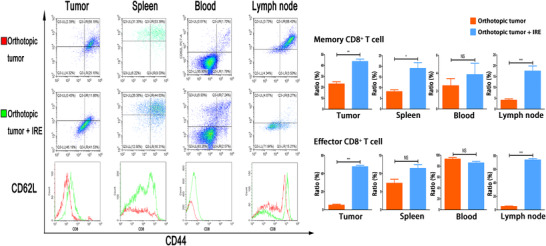
Analysis of T cells isolated from tumor, spleen, blood, and lymph node, and the comparisons of associated ratios of memory and effector CD8^+^ T cells in orthotopic tumor models before and after IRE treatment. Memory CD8^+^ T cells in tumor, spleen, lymph node, and effector CD8^+^ T cells in tumor and lymph node increased significantly after IRE. Two‐tailed Student's *t* test was performed. *P < .05, **P < .01, ***P < .001, NS, not significant

Our results suggested that IRE could increase the infiltration of CD8^+^ T cells in histologic detection analyses. The numbers of representative immune cells per gram of tumor, lymph node (LN), blood, and spleen tissue are presented in Figure 4. There were significantly higher frequencies of the total CD8^+^ T cells in the IRE group than in the control group. To further analyze the changes of subtypes of the infiltrated CD4^+^ and CD8^+^ T cells, the numbers of memory and effector T cells were detected by FC. Compared with control group, significant higher frequencies of memory CD4^+^ T cells (CD4^+^ CD44+ CD62L^−^) and CD8^+^ T cells (CD8^+^ CD44^+^ CD62L^−^) in tumor and tumor‐draining LN regions were observed in the subcutaneous pancreatic tumors (Figure 4A,B). The numbers of memory T cells in LN increased more significantly compared with those in the subcutaneous tumor, while the effector CD8^+^ T cells (CD8^+^ CD44^−^ CD62L^−^) in the tumor increased more significantly in the tumor compared with those in the LN region (Figure 4B). Similar results were also obtained in orthotopic pancreatic tumors. Compared with the control group, significant higher frequencies of memory and effector CD8^+^ T cells in tumor and LN regions were observed in the IRE group (Figure 5). These results suggested that the effector CD8^+^ T cells played an important role in IRE‐mediated antitumor effect and this effect might be sensitized by the long‐term antitumor memory.

### 3.3 IRE enhanced the immune memory of T cells and induced abscopal effect

**FIGURE 6:**
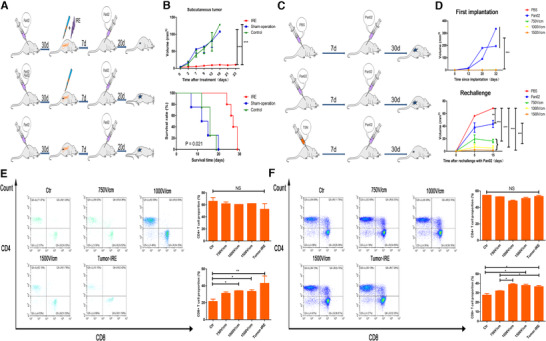
IRE enhanced the immune memory of T cells. A, Experimental protocol for the evaluation of the abscopal effect of ablation in suit by IRE. B, The comparisons of subcutaneous tumor growth curves and survival curves after the ablation by IRE in orthotopic tumor model. IRE significantly suppressed tumor growth and extended survival, compared with sham‐operation and no treatment control. C, Experimental protocol for the evaluation of the abscopal effect of tumor culture supernatants (TSN) of Pan02 3 days after electroporation with different strength levels. D, The comparisons of first‐implanted and rechallenged tumor growth curves after exposure to the TSN of Pan02 3 days after electroporation with different strength levels. Original tumors were not observed in mice, which were first implanted with TSN of Pan02 after electroporation. Additionally, the growth of rechallenged tumors decreased along with the initial treatment with TSN of Pan02 3 days after electroporation with different strength levels. E, The proportion of infiltrated CD4^+^ and CD8^+^ T cells in lymph node of mice after exposure to TSN of Pan02 3 days after electroporation with different strength levels. No significant changes were observed for CD4^+^ T cell, while the ratios of CD8^+^ T cell increased significantly along with the increasing levels of strength of electroporation. F, The proportion of infiltrated CD4^+^ and CD8^+^ T cells in spleen of mice after exposure to TSN of Pan02 3 days after electroporation with different strength levels. No significant changes were observed for CD4^+^ T cell while the ratios of CD8^+^ T cell increased significantly along with the increasing levels of strength of electroporation. Log‐rank test and one‐way analysis of variance (ANOVA) with Bonferroni comparison test were performed. *P* < .05, **P < .01, ***P < .001, NS, not significant

**FIGURE 7:**
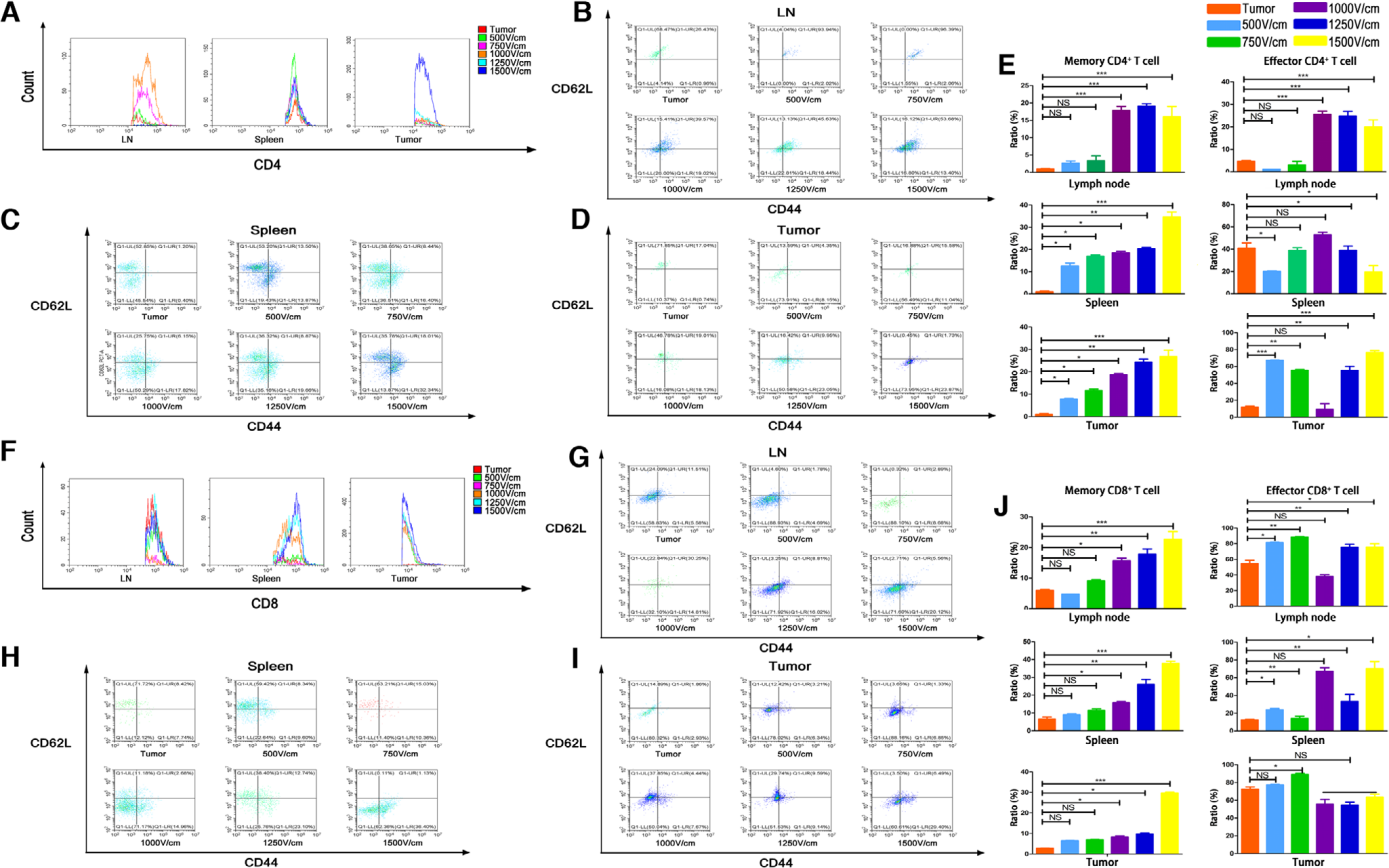
A, The comparisons of proportions of infiltrated CD4+ T cells in lymph node, spleen, and tumor of mice after exposure to TSN of Pan02 3 days after electroporation with different strength levels. No significant changes were observed for CD4^+^ T cell after exposure to the TSN of Pan02 3 days after electroporation with different strength levels. B, The proportion of infiltrated memory and effector CD4^+^ T cells in lymph node of mice after exposure to TSN of Pan02 3 days after electroporation with different strength levels. C, The proportion of infiltrated memory and effector CD4^+^ T cells in spleen of mice after exposure to TSN of Pan02 3 days after electroporation with different strength levels. D, The proportion of infiltrated memory and effector CD4^+^ T cells in tumor of mice after exposure to TSN of Pan02 3 days after electroporation with different strength levels. E, The comparisons of associated ratios of memory and effector CD4^+^ T cells in lymph node, spleen, and tumor along with the increasing strength of electroporation applied to Pan02 cells. The ratios of memory CD4^+^ T cells increased significantly in lymph node, spleen, and tumor, while the ratios of memory CD4^+^ T cells increased significantly in lymph node and tumor along with the increasing strength of electroporation. F, The comparisons of proportions of infiltrated CD8^+^ T cells in lymph node, spleen, and tumor of mice after exposure to TSN of Pan02 3 days after electroporation with different strength levels. Significant changes were observed for CD8^+^ T cell in spleen and tumor after exposure to the TSN of Pan02 3 days after electroporation with different strength levels. G, The proportion of infiltrated memory and effector CD8^+^ T cells in lymph node of mice after exposure to TSN of Pan02 3 days after electroporation with different strength levels. H, The proportion of infiltrated memory and effector CD8^+^ T cells in spleen of mice after exposure to TSN of Pan02 3 days after electroporation with different strength levels. I, The proportion of infiltrated memory and effector CD8^+^ T cells in tumor of mice after exposure to TSN of Pan02 3 days after electroporation with different strength levels. J, The comparisons of associated ratios of memory and effector CD8^+^ T cells in lymph node, spleen, and tumor along with the increasing strength of electroporation applied to Pan02 cells. The ratios of memory CD8^+^ T cells increased significantly in lymph node, spleen, and tumor, while the ratios of effector CD8^+^ T cells increased significantly in lymph node and spleen along with the increasing strength of electroporation. One‐way analysis of variance (ANOVA) with Bonferroni comparison test was performed. *P* < .05, **P < .01, ***P < .001, NS, not significant

To further investigate whether the induced memory can help to suppress the subsequent tumor, mice with orthotopic pancreatic tumor following IRE were rechallenged with Pan02 cells by percutaneous inoculation. Age‐matched mice with orthotopic pancreatic tumors following sham operation or no treatments were used as controls (Figure 6A). Mice with orthotopic pancreatic tumor after IRE rejected the subcutaneous tumor challenge and had a tumor‐free survival for almost 30 days. Oppositely, mice in the control groups exhibited a robust tumor growth and a significant shorter survival time compared with mice in the IRE group (Figure 6B). Moreover, to simulate the immune process in vivo, TSN of Pan02 3 days after electroporation with different strength levels was injected into the subcutaneous tissue on the left back of mice. Seven days after first implantation of TSN, these mice were rechallenged with same amount of Pan02 cells percutaneously on the right back (Figure 6C). Original tumors were not observed in mice which were first implanted with TSN of Pan02 after electroporation. Additionally, it was shown that the growth of rechallenged tumors decreased along with the increasing field strength of electroporation applied to the TSN of Pan02 cells. The mice after the first implantation of TSN following IRE (1500 V/cm) rejected the subcutaneous tumor challenge and were tumor‐free survival through the whole duration of the study (Figure 6D). Additionally, an underlining immunologic cause for the abscopal results obtained was reinforced by results of FC. The numbers of infiltrated CD8^+^ T cells increased gradually as the levels of electric field strength increased while no significant changes were observed for CD4^+^ T cells. The proportions of CD8^+^ T cells in mice treated with TSN following IRE was similar to those of mice treated with IRE directly (Figure 6E,F). To further analyze the system T‐cell response, subtypes of T cell from tumor‐draining LN, spleen, and tumor were detected by FC. With the increase in electric field strength, the proportions of memory CD4^+^ T cells increased gradually. The changes were more significant in the LN and tumor regions, compared with those in the spleen. The numbers of effector and memory CD8^+^ T cells were also increased in electric field strength's manners. The increase in effector ones occupied a major part in the changes of all CD8^+^ T cells (Figure 7).

### 3.4 IRE induced ICD of tumor cells

**FIGURE 8:**
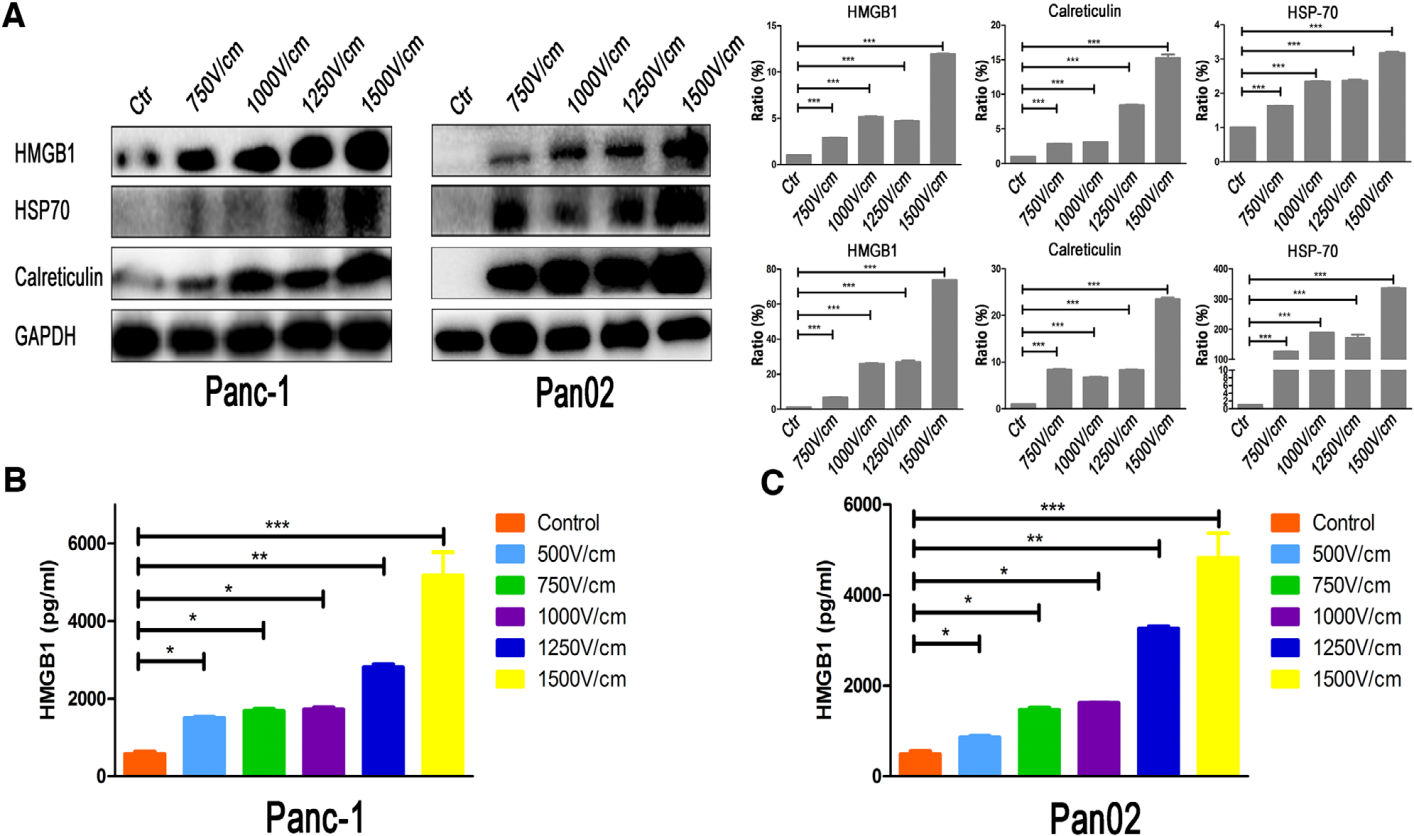
IRE induces ICD of tumor cells. A, The expression and the quantification of integrated optical density of the protein bands of intracellular DAMPs of Panc‐1 and Pan02 treated by electric fields with different field strengths. The expression of HMGB1, HSP70, and calreticulin increased significantly along with the increasing strength of electroporation. B, The analysis of released HMGB1 in TSN of Panc‐1 treated with electric fields with different field strengths by enzyme‐linked immunosorbent assay (ELISA). C, The analysis of released HMGB1 in TSN of Pan02 treated with electric fields with different field strengths by ELISA. The levels of secreted HMGB1 in TSN of Panc‐1 and Pcan02 increased significantly along with the increasing strength of electroporation. One‐way analysis of variance (ANOVA) with Bonferroni comparison test was performed. *P < .05, **P <.01, ***P < .001

IRE induced and enhanced the immune memory against tumor, which played a major role in the consistent inhibition of tumor growth. To investigate the mechanisms of potential immune effects induced by IRE, we continually evaluated the model of cell death induced by IRE in vitro. A pilot of study indicated that the expression of HMGB1, calreticulin, and HSP70 in tumor cells increased significantly as the levels of electric field strength increased (Figure 8A). The supernatants of IRE‐treated cells were analyzed for HMGB1. IRE at 1500 V/cm increased the extracellular HMGB1 concentration by 15 times in Panc‐1 cells and 17 times in Pan02 cells compared with those of control groups (Figure 8B,C). These results indicated that IRE induced cell death consistent with ICD.

### 3.5 IRE modulated stroma of TME of PDAC

**FIGURE 9:**
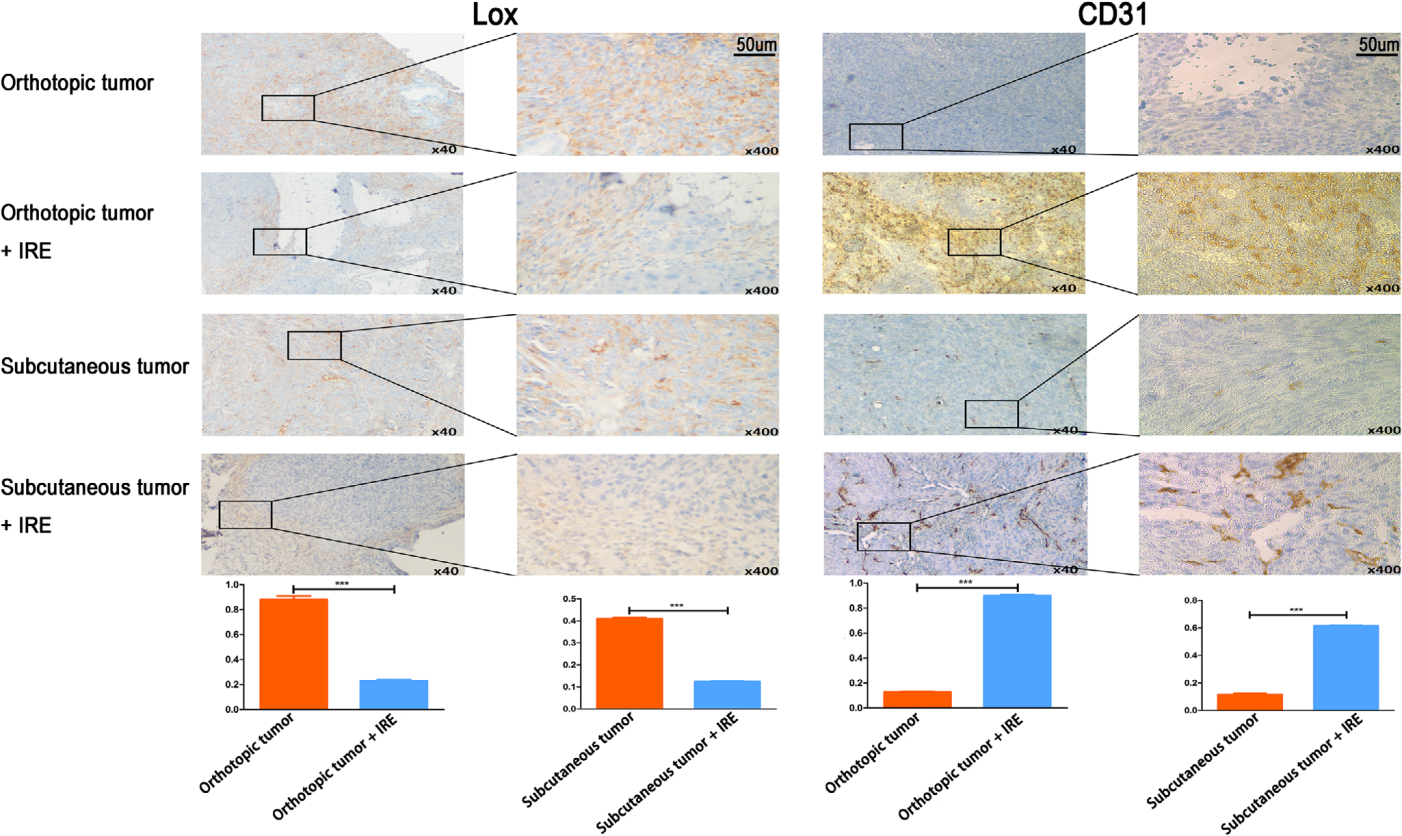
The expression of Lox and CD31 in the tumor tissue before and after IRE in orthotopic and subcutaneous pancreatic tumor models. Obvious modulation of stroma following IRE is evident from decreased Lox and increased CD31 expression in tumor of the orthotopic and subcutaneous tumor models. Two‐tailed Student's *t*‐test was performed. ***P < 0.001

Given that IRE induced an increase in CD8^+^ T cells, we speculated that the modulation of stroma might play a role in this process. IHC staining in the viable region revealed that CD31 (a marker of blood vessels) expression increased following IRE compared to untreated control, indicating that the microvessel density (MVD) increased after IRE in the tumor region. Moreover, the expression of LOX, a marker for the rigidity of the extracellular matrix decreased significantly in the viable region after IRE compared to the untreated control (Figure 9). Taken together, these findings indicated that the stroma of PDAC could be moderated by IRE by increasing MVD and softening extracellular matrix and these changes would lead to the increase in the infiltration of CD8^+^ T cells.

## 4 DISCUSSION

Previous studies have illustrated the immunomodulatory effect of IRE in PDAC patients, showing that higher proportions of CD8^+^ T cells in blood were observed after IRE.^22^, ^36^ However, the immunohistology evidences of T cells infiltration and the associated functional changes of infiltrated T cells were still lacking so far. In this study, we showed that IRE significantly suppressed tumor growth and prolonged survival time of immunocompetent mice bearing well‐established subcutaneous and orthotopic tumor. Remarkably, the ablation of PDAC in suit or exposure to TSN of tumor cells after IRE in vitro also resulted in a decreased size for remote tumors. Our results demonstrated that IRE not only increased the tumor‐infiltrated CD8^+^ T cells, which acted as a key contributor to the superior antitumor efficacy in suit, but also enhanced the immune memory by elevating the proportion of memory CD4^+^ and CD8^+^ T cells. The release of tumor antigens and the induction of pro‐inflammatory effect of IRE may contribute to the enhancement of immune memory and the induction of abscopal effect.

In our previous studies of LAPC patients,^14^, ^15^ we were interested to show the antitumor effects of IRE, which reduced growth rates of tumors, and contributed to the significant extended survival for these patients. Similar results were also observed in the orthotopic and subcutaneous pancreatic tumor models. The killing effects, which were based on defects of cell membrane, increased gradually along with the increasing levels of electric field strength, and IRE provided the strongest direct killing effect against tumor. Moreover, the antitumor effects of IRE were accompanied by an increase of CD8^+^ T cells, not only in the peripheral blood^24^ but also in the TME. Unlike surgical resection, IRE‐treated tumor cells in suit were taken up by antigen‐presenting cells (APCs), further activating immune reaction and increasing the infiltration of immune cells.

Apart from the direct killing of tumor cells by IRE, the apoptosis of tumor cells is accompanied with the expression or release of DAMPs, including HMGB1, calreticulin, and HSP70, illustrating that IRE can also induce ICD of tumor cells.^29^ Previous studies had shown that dendritic cells could be activated by DAMPs^37^, ^38^ and transported the tumor fragments to TME or draining LNs where adaptive immune reaction could take place, meaning that IRE could serve to achieve in suit tumor vaccination.^39^ To illustrate the induced specific antitumor immunity after IRE, immunocompetent mice were grafted with subcutaneous tumors 7 days after in situ ablation of orthotopic pancreatic tumor with IRE or exposure to TSN of IRE‐treated tumor cells in vitro. The local lesion treated by IRE was associated with distant immune response against tumor. Additionally, this abscopal effect was exhibited as a linear increase along with the increasing levels of strength for electric field that was used to treat Pan02 cells in vitro. These two designs of different simulation suggested the abscopal effect induced by IRE could result in a regression of distant lesions mediated by cytotoxic T‐cell response, in which the released DAMPs might played an important role. As a kind of immunogenicity, the abscopal effect means the ability to elicit an immune response, which is mainly affected by two factors: antigenicity and adjuvanticity. The former is referred to the expression of mutated or ectopic proteins released from tumor after IRE and the latter is mainly from the activation of the acquired and innate immune system, which could be activated by DAMPs.^40^ Compared with temperature‐related methods, IRE causes cell membrane rupture and induces oncolysis with less drastic alterations in protein conformation and tissue architecture,^33^, ^41^ ^,^ enhancing the antigenicity and adjuvanticity and eventually inducing a strong immune response and memory.

Previous studies have shown IRE could change the proportions of intratumoural immune effectors and reset the TME of PDAC from an immunosuppressive state, which largely excludes invading immune cells to one that is more immune activated.^33^ Compared with the decrease in immunosuppressive immune cells, such as Tregs and MDSCs,^22^, ^24^ this transformation is more relied on the increased infiltration of immune‐activated cells, including CD4^+^ T cells, CD8^+^ T cells, and APCs. ICD triggered by IRE influenced the above transformation through the following mechanisms. First, released DAMPs contributed to the maturation of APCs, enabling APC‐mediated priming or re‐stimulation of T cells locally within the tumor or tumor‐draining LNs after IRE, which was extremely helpful for the increase in effector and memory CD4^+^ and CD8^+^ T cells.^42^ Moreover, it was illustrated that the increase in proportions of effector and memory CD4^+^ and CD8^+^ T cells was not only in the tumor or tumor‐draining LNs, but also in spleen, showing that IRE increased the systematic infiltration of immune‐activated cells. The cascade of chemotaxis, with DAMPs acting on APCs and type I interferon‐induced chemokines acting on T lymphocytes, involved in the activation and recruitment of T cells,^28^, ^43^ eventually enhancing the immune response and memory. Second, local oncolysis of ICD might disrupt the tissue barriers that physically block tumor infiltration by immune‐activated cells through affecting the cellular composition of the immune infiltrate.^44^ Additionally, similar with other study,^33^ it was shown that IRE led to stromal depletion by inhibiting LOX expression, which produced a fibrotic network and restricted penetration of T cells^45^, ^46^ in this study. Our results also showed that the MVD increased after IRE, indicating that not only large vessels but also microvessels were preserved after IRE. The increase in density and permeability of microvessels in tumor region after IRE would surely facilitate the recruitment of immune‐activated cells into the tumor bed. As illustrated in this study, the immune activation induced by the increased infiltration of memory and effector T cells after IRE suppressed the growth of tumors significantly. A reverse proof of the tumor‐suppressive effect from the depletion of T cells is also needed for a better understanding of immunomodulation induced by IRE.

## 5 CONCLUSION

In conclusion, we demonstrated that IRE associated with local immunomodulation increased specific T cells infiltration and immune response. Additionally, IRE, through enhancing specific immune memory, not only led a complete tumor regression in suit, but also induced abscopal effect and suppressed the growth of the latent lesions. Given the immune‐active nature of IRE, the combination use of IRE and immunotherapy in PDAC is therefore considerable.

## AUTHORS’ CONTRIBUTIONS

Literature search and study design: He, Huang, Zhang, and Li. Data analysis: He, Huang, and Zhang. Data collection: He, Huang, Zhang, and Lin. Manuscript writing: He, Huang, and Zhang. Suggestion: He, Huang, Zhang, and Li. All authors read and approved the final manuscript.

## FUNDING INFORMATION

National Natural Science Funds; Grant Numbers: 81972299 and 81672390; National Key Research and Development Plan; Grant Number: 2017YFC0910002.

## ETHICAL APPROVAL

This animal study was reviewed and approved by Subcommittee on Research Animal Care of the Sun Yat‐sen University Cancer Center.

## CONFLICT OF INTEREST

The authors declare that they have no conflict of interest.

## Supporting information

Supporting Information

## Data Availability

The datasets generated and/or analyzed during the current study are not publicly available due personal information involved but are available from the corresponding author on reasonable request.
