# Supplementary figures and images for: T‐cell activation and immune memory enhancement induced by irreversible electroporation in pancreatic cancer
Source: Clin Transl Med. 2020 Jun 4;10(2):e39. doi: 10.1002/ctm2.39 (PMC7403705; doi:10.1002/ctm2.39)

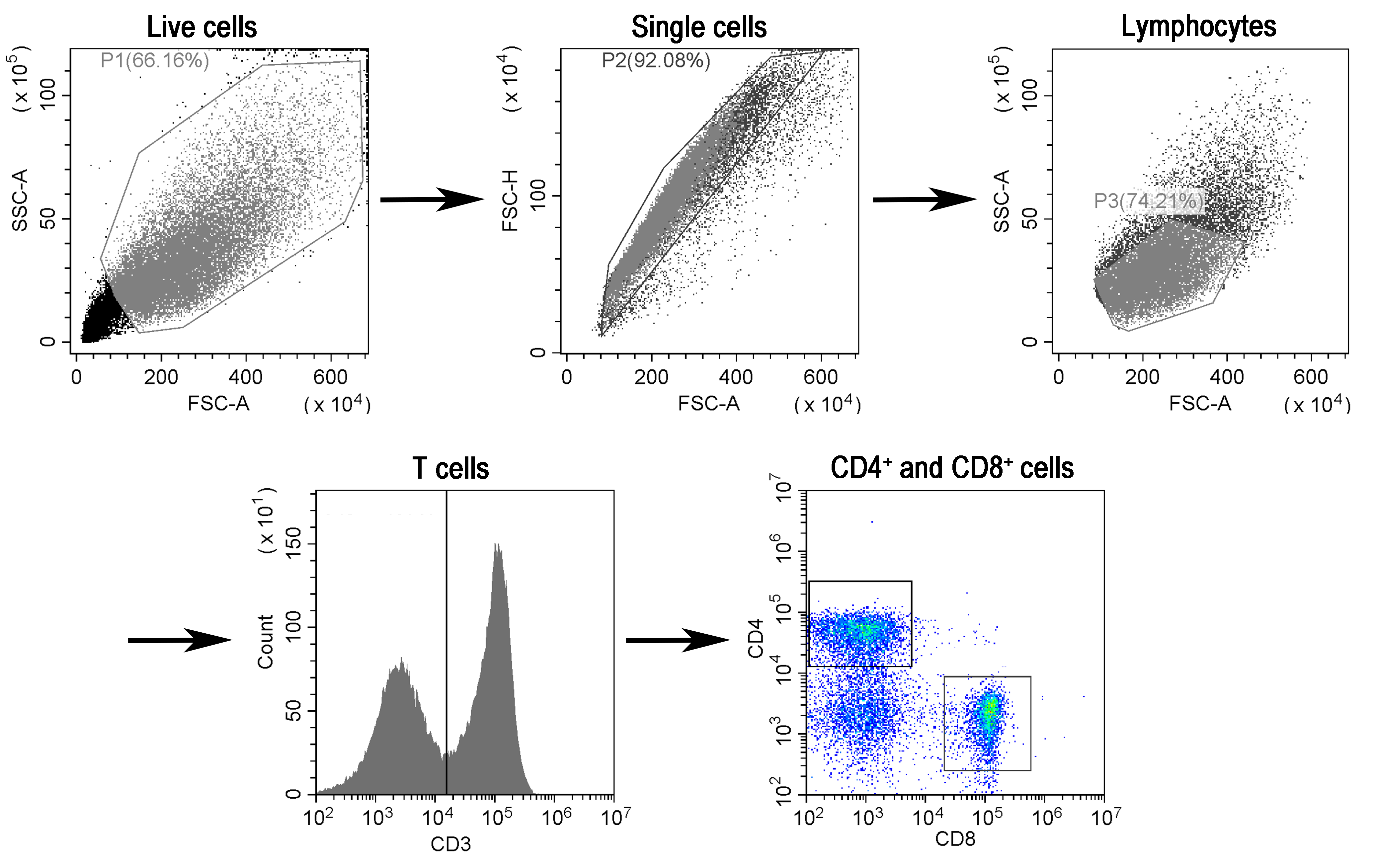

Supplement: Supplementary file 1 — Supporting Information [file CTM2-10-e39-s001.tif]
